# Supplementary material for: Structural Basis for Agonistic Activity and Selectivity toward Melatonin Receptors hMT1 and hMT2
Source: Int J Mol Sci. 2023 Feb 2;24(3):2863. doi: 10.3390/ijms24032863 (PMC9918025; doi:10.3390/ijms24032863)
Supplement: Supplementary file 1 [file ijms-24-02863-s001.zip › ijms-2189484-supplementary.pdf]

Final orthosteric site of *h*MT1

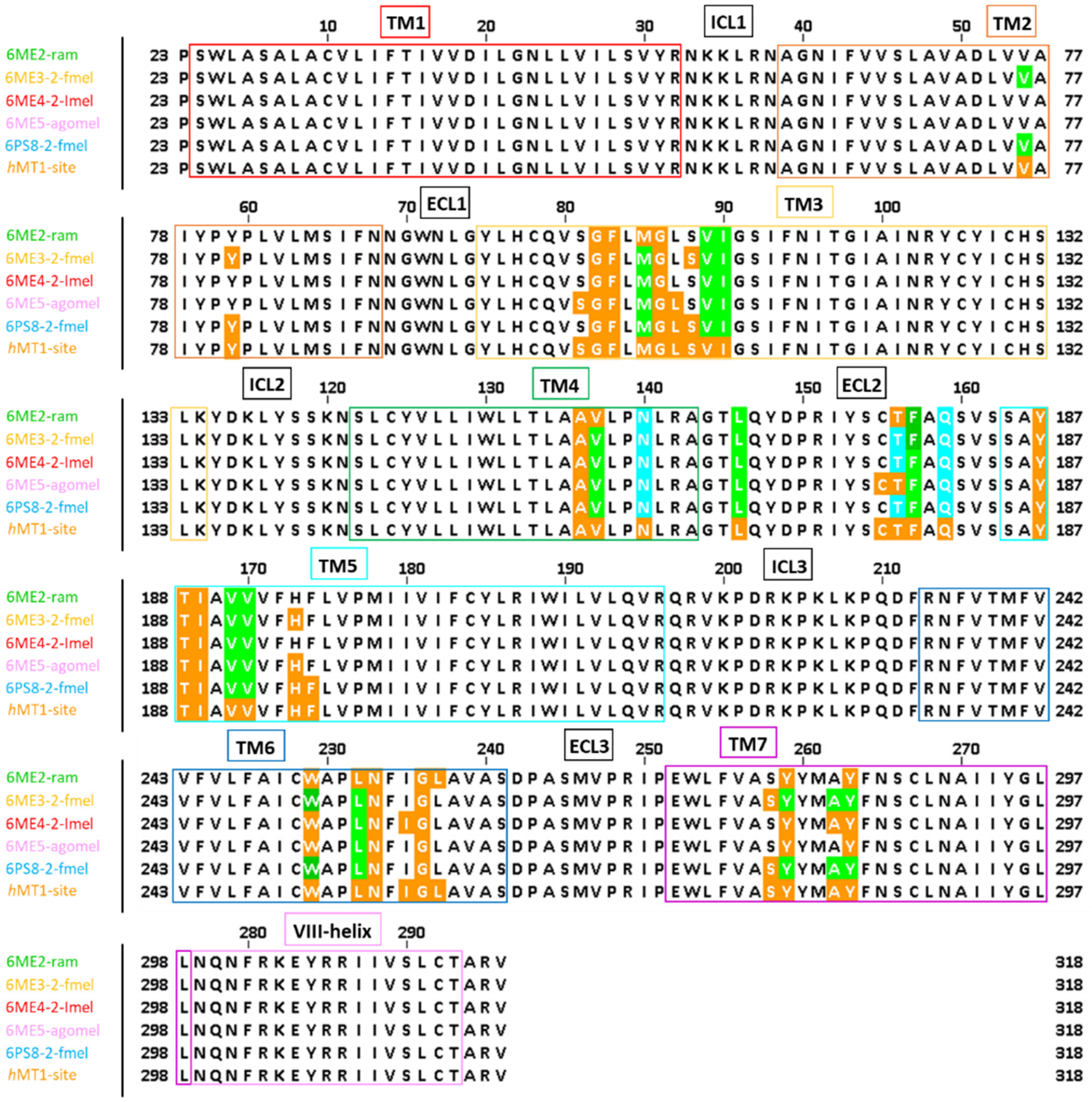

Final orthosteric site of *h*MT2

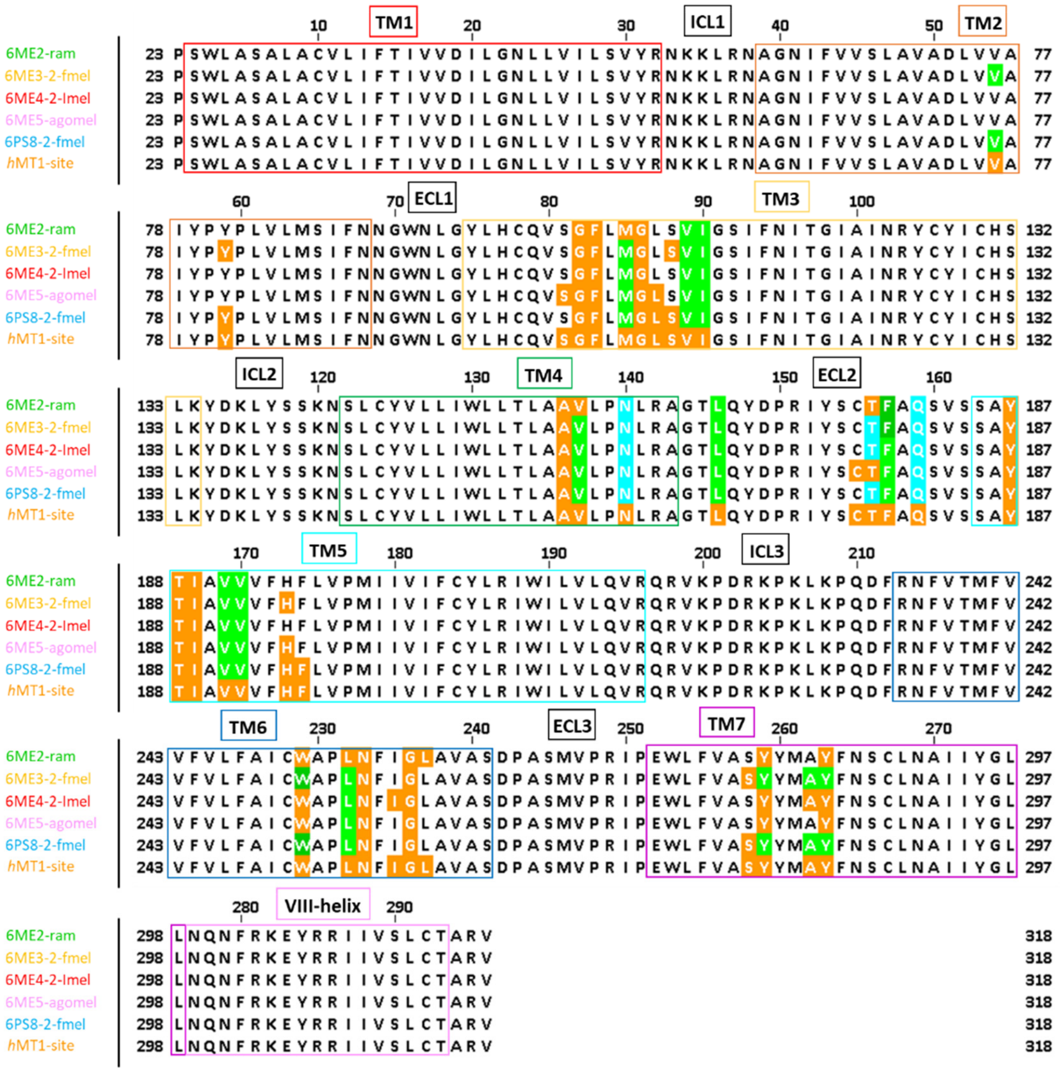

Figure S1. the amino acidic patterns of *h*MTs that participate to the intermolecular interactions.

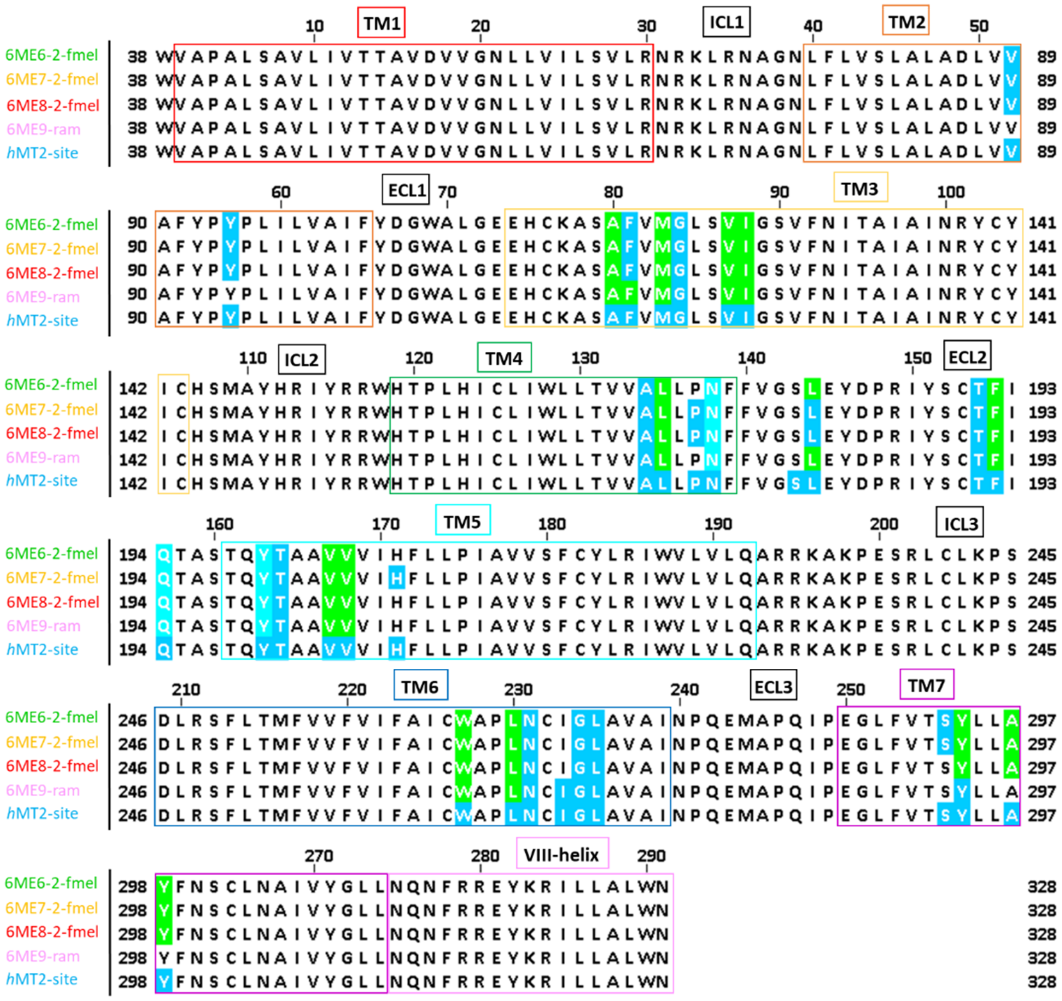

Figure S2. protein domains were squared to highlight where are localized the retrieved othosteric site residues

.

*h*MT1	*h*MT2
TM2: V76$^{2.53}$ Y81$^{2.58}$	TM2: V89$^{2.53}$ Y94$^{2.58}$
TM3: S103$^{3.28}$ G104$^{3.29}$ F105$^{3.30}$ M107$^{3.32}$ G108$^{3.33}$ L109$^{3.34}$ S110$^{3.35}$ V111$^{3.36}$ I112$^{3.37}$	TM3: A117$^{3.29}$ F118$^{3.30}$ M120$^{3.32}$ G121$^{3.33}$ V124$^{3.36}$ I125$^{3.37}$
TM4: A158$^{4.56}$ V159$^{4.57}$ N162$^{4.60}$	TM4: A171$^{4.56}$ L172$^{4.57}$ N175$^{4.60}$
ECL2: L168^{ECL2} C177 ^{ECL2} T178^{ECL2} F179^{ECL2} Q181^{ECL2}	ECL2: L181^{ECL2} T191^{ECL2} F192^{ECL2} Q194^{ECL2}
TM5: Y187$^{5.38}$ T188$^{5.39}$ I189$^{5.40}$ V191$^{5.42}$ V192$^{5.43}$ H195$^{5.46}$ F196$^{5.47}$	TM5: Y200$^{5.38}$ T201$^{5.39}$ V204$^{5.42}$ V205$^{5.43}$
TM6: W251$^{6.48}$ L254$^{6.51}$ N255$^{6.52}$ I257$^{6.54}$ G258$^{6.55}$ L259$^{6.56}$	TM6: W264$^{6.48}$ L267$^{6.51}$ N268$^{6.52}$ I270$^{6.54}$ G271$^{6.55}$ L272$^{6.56}$
TM7: S280$^{7.38}$ Y281$^{7.39}$ A284$^{7.42}$ Y285$^{7.43}$	TM7: S293$^{7.38}$ Y294$^{7.39}$ A297$^{7.42}$ Y298$^{7.43}$
**Res. 50:** N45$^{1.50}$ D73$^{2.50}$ R125$^{3.50}$ W152$^{4.50}$ P199$^{5.50}$ P253$^{6.50}$ A292$^{7.50}$	**Res. 50:** N58$^{1.50}$ D86$^{2.50}$ R138$^{3.50}$ W165$^{4.50}$ P212$^{5.50}$ P266$^{6.50}$ A305$^{7.50}$

Table S1. The table below collects all amino acids involved in stabilizing interactions; the Ballesteros–Weinstein numbering scheme for *h*MTs is also reported

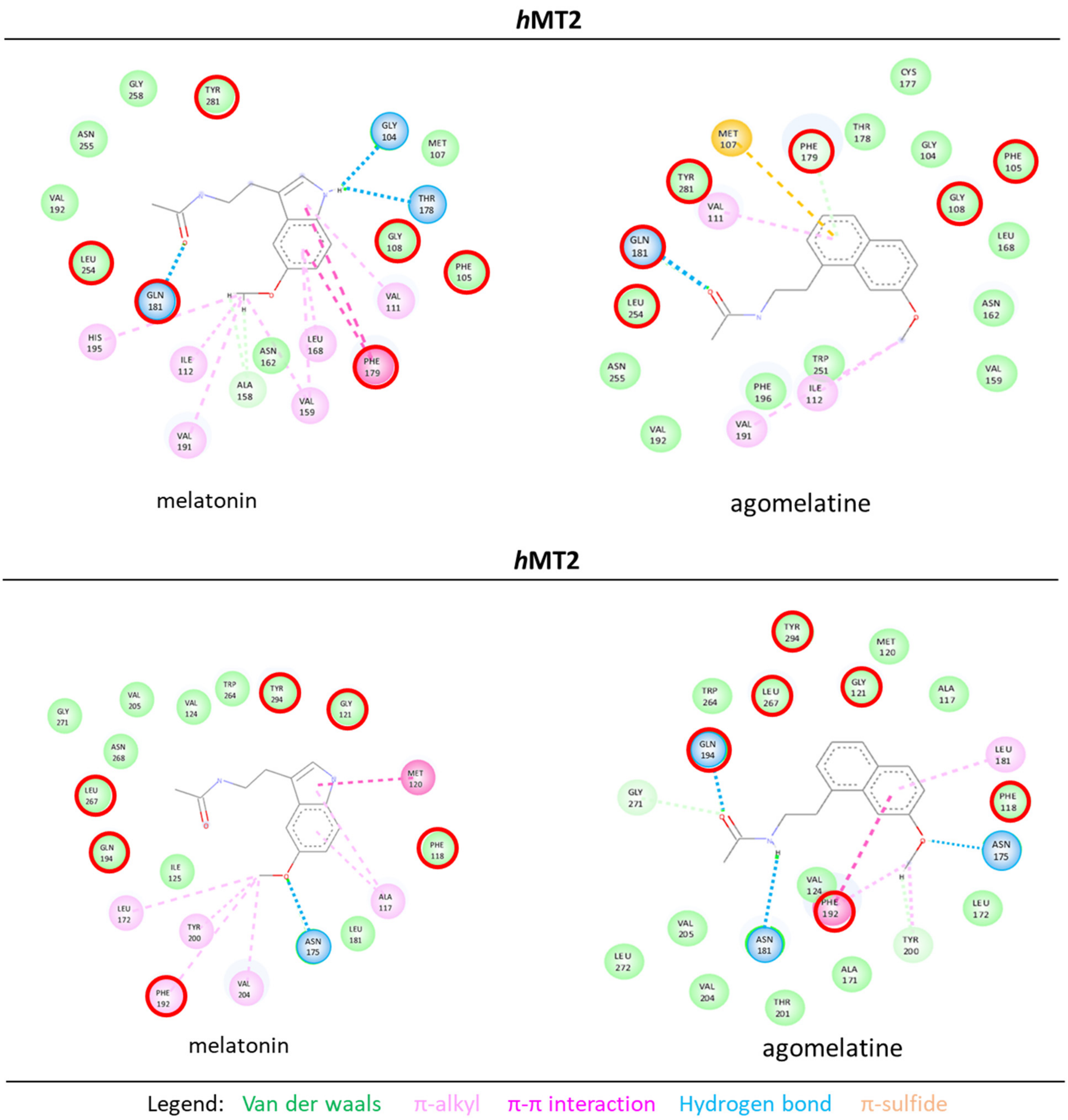

Figure S3. 2D Representation of the final poses of melatonin and agomelatine at the orthosteric site of hMT1 and hMT2, according to molecular dynamics simulations

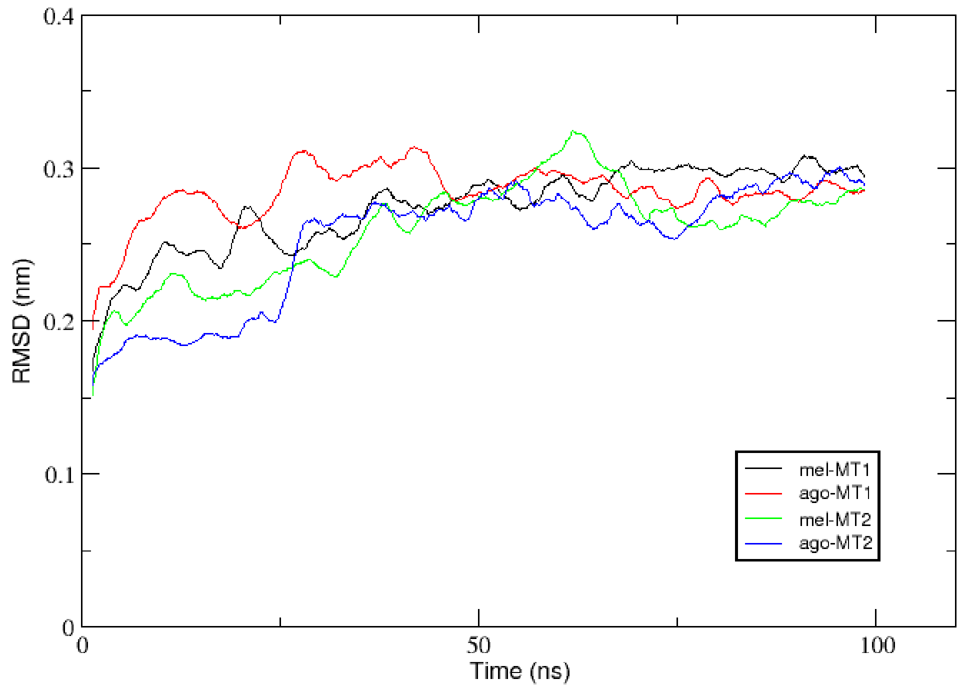

Figure S4. RMSD plot as function of time for the protein not considering hydrogen atoms. Each colour represents a different protein-ligand complex.

Receptor	Ligand	RMSD
*h*MT1	Melatonin	0.0901 nm
	Agomelatine	0.1039 nm
*h*MT2	Melatonin	0.1054 nm
	Agomelatine	0.1187 nm

Table S2. Calculation of RMSD from starting and final point of molecular dynamics simulations, using UCSF Chimera[65].

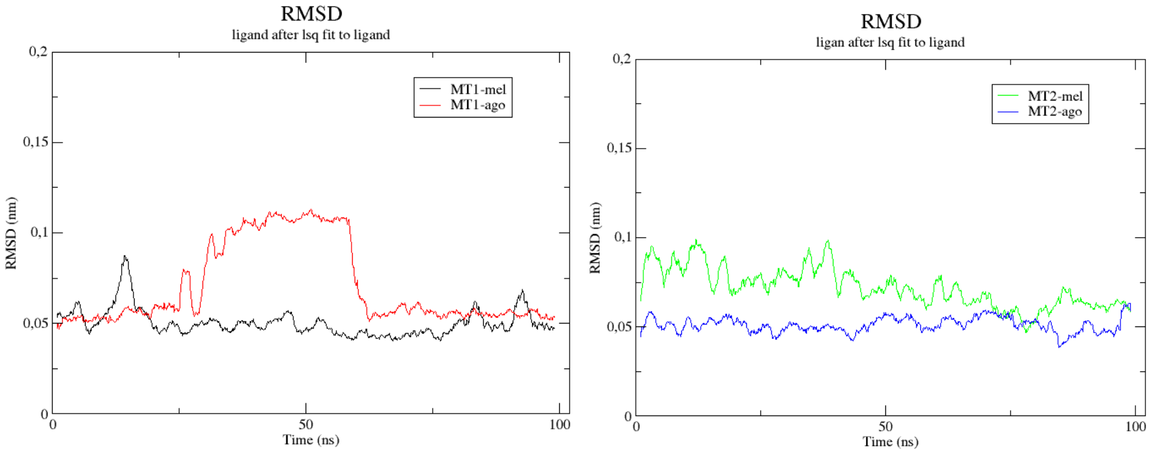

Figure S5. Time-RMSD plots for ligands in *h*MT1 and *h*MT2.
